# Modeling Group-Level Repeated Measurements of Neuroimaging Data Using the Univariate General Linear Model

**DOI:** 10.3389/fnins.2019.00352

**Published:** 2019-04-17

**Authors:** Martyn McFarquhar

**Affiliations:** Division of Neuroscience & Experimental Psychology, University of Manchester, Manchester, United Kingdom

**Keywords:** repeated measurements, within-subject, flexible factorial, SPM, FSL, GLM

## Abstract

Group-level repeated measurements are common in neuroimaging, yet their analysis remains complex. Although a variety of specialized tools now exist, it is surprising that to-date there has been no clear discussion of how repeated-measurements can be analyzed appropriately using the standard general linear model approach, as implemented in software such as SPM and FSL. This is particularly surprising given that these implementations necessitate the use of multiple models, even for seemingly conventional analyses, and that without care it is very easy to specify contrasts that do not correctly test the effects of interest. Despite this, interest in fitting these types of models using conventional tools has been growing in the neuroimaging community. As such it has become even more important to elucidate the correct means of doing so. To begin, this paper will discuss the key concept of the *expected mean squares* (EMS) for defining suitable *F*-ratios for testing hypotheses. Once this is understood, the logic of specifying correct repeated measurements models in the GLM should be clear. The ancillary issue of specifying suitable contrast weights in these designs will also be discussed, providing a complimentary perspective on the EMS. A set of steps will then be given alongside an example of specifying a 3-way repeated-measures ANOVA in SPM. Equivalency of the results compared to other statistical software will be demonstrated. Additional issues, such as the inclusion of continuous covariates and the assumption of sphericity, will also be discussed. The hope is that this paper will provide some clarity on this confusing topic, giving researchers the confidence to correctly specify these forms of models within traditional neuroimaging analysis tools.

## 1. Introduction

The modeling of group-level repeated measurements is a common, yet complex, topic in neuroimaging. Although a variety of specialized tools are now available (e.g., Chen et al., 2014; Guillaume et al., 2014; McFarquhar et al., 2016), it is surprising that to-date there has been no clear discussion of how researchers can analyse repeated measurements using the traditional voxel-wise general linear model (GLM) approach, implemented in software such as SPM (http://www.fil.ion.ucl.ac.uk) and FSL (https://fsl.fmrib.ox.ac.uk)^1^. Despite the implication from some authors (e.g., McLaren et al., 2011; Chen et al., 2014), the analysis of complex repeated-measurement designs *is* possible within these software packages. However, because they have often not been designed with these analyses in mind, specification of the correct models can be difficult. For instance, the traditional modeling of repeated measurements requires the inclusion of subject and all possible interactions with subject as factors in the model, as well as specifying multiple models to force the use of appropriate error terms for the *F*-ratios. Ignorance of the correct way to model these effects would, at best, lead to an analysis that lacked sensitivity, but at worst would lead to an analysis with an increased Type I error rate and *F*-ratios that do not test the intended model effects.

For researchers who are less familiar with classical linear model theory, some of the requirements of repeated-measurement models can seem esoteric. However, these models are based on the key statistical concept of *expected mean squares* (EMS) which, once understood, should make the logic behind these methods clear. Given the importance of the EMS for understanding the logic of hypothesis tests in repeated measures models, this paper will begin with a detailed exposition of the concept. No claims of originality are made on this exposition as these are issues well-known in the statistical literature. However, the degree of confusion surrounding these models in the neuroimaging literature has prompted an explicit discussion of this core statistical concept. An ancillary issues, in the form of *estimable functions* in overparameterized ANOVA models, will also be discussed. Once these concepts are understood, it should become clear how these models need to be treated in neuroimaging software. To that end, a set of simple steps will be given alongside an example of how to specify a 3-way repeated-measures ANOVA model in SPM12. Some further issues with repeated measurement models, such as the inclusion of continuous covariates and the assumption of sphericity, will also be discussed. The hope is that these discussions will provide some clarity on this complex topic, giving researchers the knowledge and understanding needed to confidently use these models within familiar software packages.

## 2. EMS in ANOVA Models

In order to begin understanding the requirements of repeated measurement models when implemented in the GLM, the concept of EMS in ANOVA models must be understood. A basic aim of any ANOVA model is to split the data into different sources of variation. These sources of variation are formalized in terms of the calculation of *sums-of-squares* for each model component, which are converted to *mean squares* using the degrees of freedom of the model terms. The *F*-ratios are then formed by dividing a suitable mean square for the effect of interest by a suitable mean square for the error. In order to understand the logic of these *F*-ratios, it is necessary to consider the expected value for the mean squares. The EMS represents the theoretical mean of the sampling distribution of each of the mean squares and take the form of the addition of several sources of variation. In order to test a specific effect, the *F*-ratio should be formed from two terms whose EMS differ only by the source of variation associated with that effect. Under the null hypothesis that the effect of interest is 0, the magnitudes of the two mean squares should be similar and the *F*-ratio should be close to 1. As such, the larger the discrepancy between the mean squares the larger the *F*-ratio and the greater the evidence against the null. This logic of constructing ANOVA tests is central to the ANOVA methodology, but is also one of the major sources of misunderstanding when attempting to construct tests of effects in repeated-measurement models. As such, this first section aims to describe the derivation of the EMS and their use in forming meaningful hypothesis tests.

### 2.1. EMS in Between-Subject Designs

To understand the use of EMS in deriving suitable *F*-ratios, consider a balanced two-way between-subjects ANOVA model with *n* observations per-cell

(1) $$\begin{array}{l} y_{ijk}=\mu +\alpha_{i}+\beta_{j}+{\left(\alpha \beta \right)}_{ij}+\epsilon_{ijk} \end{array}$$

where μ is the overall mean, α_*i*_ is the effect of the *i*th level of factor A (*i* = 1, …, *a*), β_*j*_ is the *j*th level of factor B (*j* = 1, …, *b*), (αβ)_*ij*_ is the *ij* interaction effect and ϵ_*ijk*_ is random error (*k* = 1, …, *n*) assumed $\epsilon_{ijk}\sim N\left(0,\sigma^{2}\right)$.

For this basic ANOVA design, the correct error term for the omnibus main effects and interaction is the model variance σ^2^. To see why this is the case, we can calculate the EMS. Although possible to derive the EMS formally through the use of the expectation operator, a more practical approach involves following some basic rules. In this paper, the rules given by Kutner et al. (2004) are used. As an example, Table 1 gives an outline of the arithmetic involved in constructing the EMS for the model in Equation (1). These tables are provided throughout this paper to give direct correspondence between the method of Kutner et al. (2004) and the eventual forms of the EMS used to derive appropriate *F*-ratios.

**Table 1:** Arithmetic for the derivation of the EMS in a 2-way between-subjects ANOVA model, using the method of Kutner et al. (2004).

	**i**	**j**	**k**					
	**F**	**F**	**R**		**EMS_***A***_**	**EMS_***B***_**	**EMS_***AB***_**	**EMS_***E***_**
	**a**	**b**	**n**	**Variance**	**i**	**j**	**ij**	***k*(*ij*)**
α_*i*_	0	*b*	*n*	$\sigma_{\alpha }^{2}$	*bn*	0	0	0
β_*j*_	*a*	0	*n*	$\sigma_{\beta }^{2}$	0	*an*	0	0
(αβ)_*ij*_	0	0	*n*	$\sigma_{\alpha \beta }^{2}$	0	0	*n*	0
ϵ_*k*(*ij*)_	1	1	1	σ^2^	1	1	1	1

Using Table 1, the EMS for the terms in Equation (1) are given in Equation (2). Construction of an appropriate *F*-ratio involves using the EMS to identify two mean squares which differ only by the effect of interest. For instance, to test the effect of factor A we must identify which EMS in Equation (2) differs only from EMS_*A*_ by $bn\frac{\sum \alpha_{i}^{2}}{a-1}$. In this instance, the only choice is EMS_*E*_. As such, a test for the effect of A can be constructed using the ratio of the mean square of A (MS_*A*_) and the mean square of the errors (MS_*E*_). Continuing in this fashion, suitable *F*-ratios for all the model effects can be derived, as given in Table 2. This confirms the initial statement that a suitable denominator for all tests from the model in Equation (1) is the overall error term.

(2) $$\begin{array}{lll} {\text{EMS}}_{A} & = & \sigma^{2}+bn\frac{\sum \alpha_{i}^{2}}{a-1}\\ {\text{EMS}}_{B} & = & \sigma^{2}+an\frac{\sum \beta_{j}^{2}}{b-1}\\ {\text{EMS}}_{AB} & = & \sigma^{2}+n\frac{\sum {\left(\alpha \beta \right)}_{ij}^{2}}{\left(a-1)(b-1\right)}\\ {\text{EMS}}_{E} & = & \sigma^{2} \end{array}$$

**Table 2:** The numerator and denominator mean squares from Equation (2) used to form appropriate *F*-tests for the model in Equation (1).

**Effect**	**Test**
Factor A	MS_*A*_/MS_*E*_
Factor B	MS_*B*_/MS_*E*_
A × B	MS_*AB*_/MS_*E*_

### 2.2. EMS in Mixed-Measures Designs

For between-subject designs containing only fixed-effects it is rarely necessary to calculate the EMS as a suitable denominator for the *F*-ratios is always given by the overall error term. The situation becomes quite different when considering ANOVA models containing random-effects. Although not usually applicable in neuroimaging for between-subjects designs, when within-subject and mixed within-subject and between-subjects (mixed-measures) designs are considered, it is usually desirable to include *subject*, as well as all possible interactions with *subject*, as random-effects. In doing so, the structure of the EMS changes and the derivation of a suitable error term for testing hypotheses about particular effects becomes more complex.

#### 2.2.1. A Single Within-Subject Factor

To begin, consider a basic mixed-measures design containing a single within-subject factor and a single between-subjects factor. The model is

(3) $$\begin{array}{l} y_{ijk}=\mu +\alpha_{i}+\beta_{j}+{\left(\alpha \beta \right)}_{ij}+S_{k\left(j\right)}+\epsilon_{ijk} \end{array}$$

where μ is the overall mean, α_*i*_ is the effect of the *i*th level of the within-subject factor (*i* = 1, …, *a*), β_*j*_ is the effect of the *j*th level of the between-subjects factor (*j* = 1, …, *b*) and (αβ)_*ij*_ is the *ij* interaction effect. *S*_*k*(*j*)_ is the random effect of the *k*th subject (*k* = 1, …, *n*) assumed $S_{k\left(j\right)}\sim N\left(0,\sigma_{s}^{2}\right)$ and ϵ_*ijk*_ is random error assumed $\epsilon_{ijk}\sim N\left(0,\sigma^{2}\right)$. The notation *S*_*k*(*j*)_ indicates that the *k*th subject is *nested* within group *j*. This conveys the fact that, for example, *k* = 1 refers to a different subject depending on the value of *j* (see Chapter 26 in Kutner et al., 2004).

One of the key differences between the model in Equation (1) and the model in Equation (3) is the inclusion of the random subject effects *S*_*k*(*j*)_. Although possible to forego the subject effects and work with a pooled error term (Penny and Henson, 2007), doing so produces tests which are more conservative (see Casella, 2008, p. 85). As such, the inclusion of *S*_*k*(*j*)_ allows one to *partition* the model errors in order produce more sensitive tests of the model effects. Some intuition can be gained here by re-writing Equation (3) as

(4) $$\begin{array}{l} y_{ijk}=\mu +\alpha_{i}+\beta_{j}+{\left(\alpha \beta \right)}_{ij}+\epsilon_{k\left(j\right)}^{\left(1\right)}+\epsilon_{ijk}^{\left(2\right)} \end{array}$$

where the splitting of the singular error term is now more explicit. The complication for the traditional neuroimaging GLM framework is that the error term used as the denominator for the test statistics is derived implicitly from the difference between the data and the model prediction. This means that for the model in Equation (4), only $\epsilon_{ijk}^{\left(2\right)}$ will be used in the formation of the test statistics. Furthermore, in order to correctly derive the final error term, $\epsilon_{k\left(j\right)}^{\left(1\right)}$ must also be included in the design matrix, despite its status as a random-effect.

As with before, the breakdown of the arithmetic in Table 3 gives the calculation of the EMS for the mixed-measures ANOVA model given in Equation (3). The final EMS are given in Equation (5) with suitable ratios for testing the main effects and interactions given in Table 4. Of particular importance here is to recognize how both the A and A × B effects use the overall error term, but that a suitable *F*-ratio for the effect of B requires the use of MS_*S*_ as the denominator instead. As discussed above, only MS_*E*_ will be used as the denominator in neuroimaging software implementing the traditional GLM approach. Testing of the effect of B therefore requires specifying a separate model where the final error term is forced to become MS_*S*_. This can be achieved by averaging the raw data over the levels of the within-subject factor, and will be discussed in more detail in the example analysis given at the end of this paper.

(5) $$\begin{array}{lll} {\text{EMS}}_{A} & = & \sigma^{2}+bn\frac{\sum \alpha_{i}^{2}}{a-1}\\ {\text{EMS}}_{B} & = & \sigma^{2}+a\sigma_{s}^{2}+an\frac{\sum \beta_{j}^{2}}{b-1}\\ {\text{EMS}}_{AB} & = & \sigma^{2}+n\frac{\sum {\left(\alpha \beta \right)}_{ij}^{2}}{\left(a-1)(b-1\right)}\\ {\text{EMS}}_{S} & = & \sigma^{2}+a\sigma_{s}^{2}\\ {\text{EMS}}_{E} & = & \sigma^{2} \end{array}$$

**Table 3:** Arithmetic for the derivation of the EMS in a 2-way mixed ANOVA with a single within-subject and a single between-subjects factor.

	**i**	**j**	**k**						
	**F**	**F**	**R**		**EMS_***A***_**	**EMS_***B***_**	**EMS_***AB***_**	**EMS_***S***_**	**EMS_***E***_**
	**a**	**b**	**n**	**Variance**	**i**	**j**	**ij**	***k*(*j*)**	***k*(*ij*)**
α_*i*_	0	*b*	*n*	$\sigma_{\alpha }^{2}$	*bn*	0	0	0	0
β_*j*_	*a*	0	*n*	$\sigma_{\beta }^{2}$	0	*an*	0	0	0
(αβ)_*ij*_	0	0	*n*	$\sigma_{\alpha \beta }^{2}$	0	0	*n*	0	0
*S*_*k*(*j*)_	*a*	1	1	$\sigma_{s}^{2}$	0	*a*	0	*a*	0
ϵ_*k*(*ij*)_	1	1	1	σ^2^	1	1	1	1	1

**Table 4:** The EMS ratios used to form appropriate *F*-tests for the main effects and interactions in a 2-way mixed-measures ANOVA.

**Effect**	**Test**
A	MS_*A*_/MS_*E*_
B	MS_*B*_/MS_*S*_
A × B	MS_*AB*_ / MS_*E*_

#### 2.2.2. Multiple Within-Subject Factors

The situation with multiple error terms becomes more complex as the number of within-subject factors increases. Consider adding another within-subject factor to the model in Equation (3). This produces a 3-way mixed-measures ANOVA model, which can be written as

(6) $$\begin{array}{ll} y_{ijkl}=\mu & +\alpha_{i}+\beta_{j}+\gamma_{k}+{\left(\alpha \beta \right)}_{ij}+{\left(\alpha \gamma \right)}_{ik}+{\left(\beta \gamma \right)}_{jk}\\ +{\left(\alpha \beta \gamma \right)}_{ijk}+S_{l\left(k\right)}+{\left(S\alpha \right)}_{il\left(k\right)}+{\left(S\beta \right)}_{jl\left(k\right)}+\epsilon_{ijkl} \end{array}$$

where β_*j*_ is now the effect of the *j*th level of the additional within-subject factor, γ_*k*_ is the effect of the *k*th level of the between-subjects factor (*k* = 1, …, *c*) and the subject effects are indexed by *l* = 1, …, *n*. In comparison to the model in Equation (3), inclusions of additional within-subject factors provides an opportunity for further interactions with the subject effects. These are given by the interaction with the first within-subject factor (*Sα*)_*il*(*k*)_ and the interaction with the second within-subject factor (*Sβ*)_*jl*(*k*)_. Because these are interactions with a random factor, these effects are also considered random-effects and thus represent a further partitioning of the error term. Although it may initially appear as though a 3-way interaction with subject could also be included, this is not possible as it would be perfectly collinear with the errors. This is a clue to the fact that the error term in this model *is* the 3-way interaction with the subject effects.

As this is a much larger model than the previous example, the derivation of the EMS is more lengthy process. As with before, the arithmetic is presented in Table 5 and the EMS are given in Equation (7). We can see that there are now four possible error terms, given by MS_*S*_, MS_*SA*_, MS_*SB*_, and MS_*E*_, respectively. As indicated above, MS_*E*_ could equivalently be written as MS_*SAB*_ to denote the equivalence with the highest-order interaction between the subjects and within-subject factors. Suitable tests for the model effects are given in Table 6, presenting a much more complex arrangements where no more than two effects are tested using the same error term.

(7) $$\begin{array}{lll} {\text{EMS}}_{A} & = & \sigma^{2}+b\sigma_{s\alpha }^{2}+bcn\frac{\sum \alpha_{i}^{2}}{a-1}\\ {\text{EMS}}_{B} & = & \sigma^{2}+a\sigma_{s\beta }^{2}+acn\frac{\sum \beta_{j}^{2}}{b-1}\\ {\text{EMS}}_{C} & = & \sigma^{2}+ab\sigma_{s}^{2}+abn\frac{\sum \gamma_{k}^{2}}{c-1}\\ {\text{EMS}}_{AB} & = & \sigma^{2}+cn\frac{\sum {\left(\alpha \beta \right)}_{ij}^{2}}{\left(a-1)(b-1\right)}\\ {\text{EMS}}_{AC} & = & \sigma^{2}+b\sigma_{s\alpha }^{2}+bn\frac{\sum {\left(\alpha \gamma \right)}_{ik}^{2}}{\left(a-1)(c-1\right)}\\ {\text{EMS}}_{BC} & = & \sigma^{2}+a\sigma_{s\beta }^{2}+an\frac{\sum {\left(\beta \gamma \right)}_{jk}^{2}}{\left(b-1)(c-1\right)}\\ {\text{EMS}}_{ABC} & = & \sigma^{2}+n\frac{\sum {\left(\alpha \beta \gamma \right)}_{ijkl}^{2}}{\left(a-1)(b-1)(c-1\right)}\\ {\text{EMS}}_{S} & = & \sigma^{2}+ab\sigma_{s}^{2}\\ {\text{EMS}}_{SA} & = & \sigma^{2}+b\sigma_{s\alpha }^{2}\\ {\text{EMS}}_{SB} & = & \sigma^{2}+a\sigma_{s\beta }^{2}\\ {\text{EMS}}_{E} & = & \sigma^{2} \end{array}$$

**Table 5:** Arithmetic for the derivation of the EMS in the 3-way mixed-measures ANOVA with two within-subject and one between-subjects factor.

	**i**	**j**	**k**	**l**												
	**F**	**F**	**F**	**R**		**EMS_***A***_**	**EMS_***B***_**	**EMS_***C***_**	**EMS_***AB***_**	**EMS_***AC***_**	**EMS_***BC***_**	**EMS_***ABC***_**	**EMS_***S***_**	**EMS_***SA***_**	**EMS_***SB***_**	**EMS_***E***_**
	**a**	**b**	**c**	**n**	**Variance**	**i**	**j**	**k**	**ij**	**ik**	**jk**	**ijk**	***l*(*k*)**	***il*(*k*)**	***jl*(*k*)**	***l*(*ijk*)**
α_*i*_	0	*b*	*c*	*n*	$\sigma_{\alpha }^{2}$	*bcn*	0	0	0	0	0	0	0	0	0	0
β_*j*_	*a*	0	*c*	*n*	$\sigma_{\beta }^{2}$	0	*acn*	0	0	0	0	0	0	0	0	0
γ_*k*_	*a*	*b*	0	*n*	$\sigma_{\gamma }^{2}$	0	0	*abn*	0	0	0	0	0	0	0	0
(αβ)_*ij*_	0	0	*c*	*n*	$\sigma_{\alpha \beta }^{2}$	0	0	0	*cn*	0	0	0	0	0	0	0
(αγ)_*ik*_	0	*b*	0	*n*	$\sigma_{\alpha \gamma }^{2}$	0	0	0	0	*bn*	0	0	0	0	0	0
(βγ)_*jk*_	*a*	0	0	*n*	$\sigma_{\beta \gamma }^{2}$	0	0	0	0	0	*an*	0	0	0	0	0
(α*βγ*)_*ijk*_	0	0	0	*n*	$\sigma_{\alpha \beta \gamma }^{2}$	0	0	0	0	0	0	*n*	0	0	0	0
*S*_*l*(*k*)_	*a*	*b*	1	1	$\sigma_{s}^{2}$	0	0	*ab*	0	0	0	0	*ab*	0	0	0
(*Sα*)_*il*(*k*)_	0	*b*	1	1	$\sigma_{S\alpha }^{2}$	*b*	0	0	0	*b*	0	0	0	*b*	0	0
(*Sβ*)_*jl*(*k*)_	*a*	0	1	1	$\sigma_{S\beta }^{2}$	0	*a*	0	0	0	*a*	0	0	0	*a*	0
ϵ_*l*(*ijk*)_	1	1	1	1	σ^2^	1	1	1	1	1	1	1	1	1	1	1

**Table 6:** The EMS ratios used to form appropriate *F*-tests for the main effects and interactions in a 3-way mixed-measures ANOVA.

**Effect**	**Test**
A	MS_*A*_/MS_*SA*_
B	MS_*B*_/MS_*SB*_
C	MS_*C*_/MS_*S*_
A × B	MS_*AB*_/MS_*E*_
A × C	MS_*AC*_/MS_*SA*_
B × C	MS_*BC*_/MS_*SB*_
A × B × C	MS_*ABC*_/MS_*E*_

### 2.3. Section Summary

EMS are a necessary concept in ANOVA models in order to define suitable tests for the model effects. In purely fixed-effects models it is rarely necessary to explicitly calculate the EMS as a suitable denominator for each test is always given by the overall error term. When random effects are included, such as in repeated-measurements models with partitioned errors, complications arise in the derivation of suitable tests. As a minimum, models with a single within-subject factor have a choice of two error terms to form tests, whereas those with multiple within-subject factors have multiple possibilities when forming tests. It is precisely this issue of specifying the correct error term that leads to problems when using neuroimaging software designed to only use a single error term. Unless the EMS are taken into consideration it is entirely possible to end up with *F*-ratios that do not actually test the intended model effects. For instance, testing of MS_*B*_ from Equation (3) using MS_*E*_ would not result in a test of the between-subject effect, but a test of the between-subject effect *plus* the between-subject error, leading to an artificial inflation of the *F*-statistic (as noted previously by McLaren et al., 2011). Considering that the between-subject results are often of great interest in clinical neuroimaging studies, the ramifications of inflating these effects could be dire. Furthermore, these issues are not constrained to just between-subject effects. Considering the breakdown of the EMS in Equation (6), it is clear that the use of MS_*E*_ as the denominator of the *F*-ratios could lead to over-inflated statistics for all but the A × B and A × B × C interaction effects. As such, it is vital that the correct error terms are derived and then enforced to make sure that the tests of the model effects are accurate.

## 3. Contrast Weights

In the previous section we saw the importance of using EMS to derive suitable error terms in ANOVA models. Although this represents the core issue at the heart of implementing repeated measurement models in the GLM, it is also worth considering the practical question of how questions can be asked of these models in the form of contrast weights. Although the contrast framework is a well-established aspect of hypothesis testing in the neuroimaging GLM (e.g., Poline et al., 2007), additional complications arise when implementing repeated measurements models. This is due to both the inclusion of the subject effects in the model and the necessity of using overparameterized designs in certain software packages (such as SPM). These complications have unfortunately lead to some dubious advice on forming contrast weights in these models, which shall be discussed below. In addition, the use of contrast weights provides further insights into the topic of the EMS and formation of *F*-ratios, and so provides a complimentary perspective on the issues discussed in the previous section.

### 3.1. Contrast Weights for Overparameterized Repeated-Measurement Models

Consider the overparameterized design matrix for a 2 × 2 mixed-measures ANOVA with *n* = 2, given in Equation (9). Each row represents the linear combination of parameters which form one of the model predictions. As an example, the first row is given by

$$\begin{array}{l} {\text{X}}_{1}^{\left(A\right)}=\left[\begin{array}{ccccccccccccc} 1 & 1 & 0 & 1 & 0 & 1 & 0 & 0 & 0 & 1 & 0 & 0 & 0 \end{array}\right] \end{array}$$

which tells us the combination of parameters needed to calculate the prediction for the A1B1 cell for subject 1, defining an estimable function of the parameters (see McFarquhar, 2016). Because linear combinations of estimable functions are themselves estimable (see McCulloch et al., 2008, p. 122), the rows of the design matrix can be used as the building-blocks for deriving contrast weights, irrespective of the form that the design matrix takes. Furthermore, note that this prediction is given by

(8) $$\begin{array}{l} \mu_{111}={\text{X}}_{1}^{\left(A\right)}\beta \\ =\mu +\alpha_{1}+\beta_{1}+{\left(\alpha \beta \right)}_{11}+S_{1\left(1\right)} \end{array}$$

which is a combination of both the fixed and random model effects. Although the subject effects are not of interest, their inclusion in the design matrix means we cannot simply give them a weight of 0 when calculating cell or marginal means as this would define a non-estimable function. As such, calculation of the ANOVA effects from cell and marginal means will include the subject terms. For certain ANOVA effects, the subject terms will cancel in the numerator, whereas for others they will not. For those where they do not, an error term must be selected such that the subject effects are also present in the denominator. This is simply a re-statement of the general approach to constructing *F*-ratios using the EMS, but from the perspective of contrast weights.

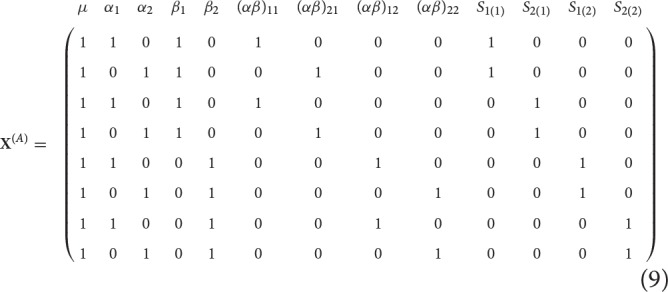



As an example, consider deriving the weights for testing the main effect of the within-subject factor A. To do so, we can first average the rows in Equation (9) which code the first level of factor A and then average the rows which code the second level of factor A^2^. This gives

(10) $$\begin{array}{l} {\text{G}}_{A1}^{\left(A\right)}=\left[\begin{array}{ccccccccccccc} 1 & 1 & 0 & 1/2 & 1/2 & 1/2 & 0 & 1/2 & 0 & 1/4 & 1/4 & 1/4 & 1/4 \end{array}\right]\\ {\text{G}}_{A2}^{\left(A\right)}=\left[\begin{array}{ccccccccccccc} 1 & 0 & 1 & 1/2 & 1/2 & 0 & 1/2 & 0 & 1/2 & 1/4 & 1/4 & 1/4 & 1/4 \end{array}\right] \end{array}$$

providing the weights for calculating the marginal means of factor A. Notice that these weights are non-zero for the subject effects. The weights for the main effect are then formed from the subtraction of the weights for the marginal means, giving

(11) $$\begin{array}{l} {\text{L}}_{A}^{\left(A\right)}={\text{G}}_{A1}^{\left(A\right)}-{\text{G}}_{A2}^{\left(A\right)} \end{array}$$

(12) $$\begin{array}{l} =\left[\begin{array}{ccccccccccccc} 0 & 1 & -1 & 0 & 0 & 1/2 & -1/2 & 1/2 & -1/2 & 0 & 0 & 0 & 0 \end{array}\right] \end{array}$$

where we can see that the subject effects have canceled. Now consider deriving the weights for testing the main effect of the between-subject factor B. Taking a similar approach to above we find

(13) $$\begin{array}{l} {\text{G}}_{B1}^{\left(A\right)}=\left[\begin{array}{ccccccccccccc} 1 & 1/2 & 1/2 & 1 & 0 & 1/2 & 1/2 & 0 & 0 & 1/2 & 1/2 & 0 & 0 \end{array}\right]\\ {\text{G}}_{B2}^{\left(A\right)}=\left[\begin{array}{ccccccccccccc} 1 & 1/2 & 1/2 & 0 & 1 & 0 & 0 & 1/2 & 1/2 & 0 & 0 & 1/2 & 1/2 \end{array}\right] \end{array}$$

which provide the weights for the marginal means of factor B which again contain non-zero weights for the subject effects. Subtracting these weights gives

(14) $$\begin{array}{l} {\text{L}}_{B}^{\left(A\right)}={\text{G}}_{B1}^{\left(A\right)}-{\text{G}}_{B2}^{\left(A\right)} \end{array}$$

(15) $$\begin{array}{l} =\left[\begin{array}{ccccccccccccc} 0 & 0 & 0 & 1 & -1 & 1/2 & 1/2 & -1/2 & -1/2 & 1/2 & 1/2 & -1/2 & -1/2 \end{array}\right] \end{array}$$

which notably is still non-zero for the subject terms. This has direct correspondence with the definitions of the EMS from earlier where EMS_*B*_ in Equation (3) contains $\sigma_{s}^{2}$. In order to form a meaningful *F*-ratio using the weights given above, one would need to select an error term that also contained the subject effects. As this is not possible by default in most neuroimaging implementations of the GLM, the use of the above contrast would be inappropriate for testing the between-subject effect of factor B. This is because, as stated earlier, the magnitude of the test-statistics would be inflated by the inclusion of the subject effects in the numerator, but not in the denominator. This speaks to a general rule-of-thumb for implementing these models in neuroimaging software, namely that appropriate contrast weights should always contain zeros for all terms containing the subject effects. Notably, this goes against the methods given by Gläscher and Gitelman (2008), where contrasts containing weights for the subject-terms are given as means of testing all the ANOVA effects within the same model. Hopefully it is now clear why this advice is inappropriate.

### 3.2. Contrast Weights for Non-overparameterized Repeated-Measurement Models

To see how the discussions in the previous section are readily applicable to non-overparameterized models (such as those used in FSL FEAT), consider the design matrices given in Equation (16). These are both constrained versions of the matrix from Equation (9), with **X**^(*B*)^ using “treatment” coding and **X**^(*C*)^ using “sigma-restricted” coding (see McFarquhar, 2016). Of note is the fact that “cell means” coding could also be used to simplify Equation (9), but that the design would remain overparameterized (although the contrast weights would be simpler).

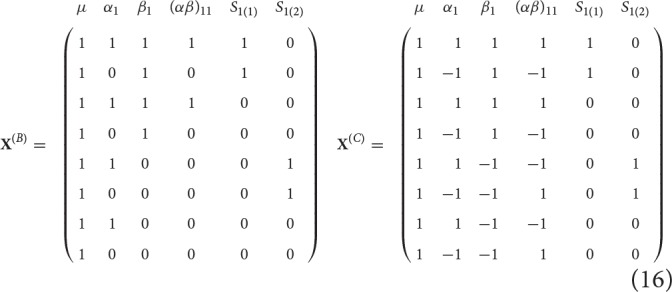



Application of the earlier approach to deriving contrasts leads to the weights for the effect of within-subject factor A in model B (${\text{L}}_{A}^{\left(B\right)}$) and model C (${\text{L}}_{A}^{\left(C\right)}$), as given in Equation (17).

(17) $$\begin{array}{llllll} G_{A1}^{\left(B\right)} & = & \left[\begin{array}{cccccc} 1 & 1 & 1/2 & \;1/2 & 1/4 & 1/4 \end{array}\right] & G_{A1}^{\left(C\right)} & = & \left[\begin{array}{llllll} 1 & \;1 & 0 & 0 & 1/2 & 1/2 \end{array}\right]\\ G_{A2}^{\left(B\right)} & = & \left[\begin{array}{cccccc} 1 & 0 & 1/2 & \;0 & \;1/4 & 1/4 \end{array}\right] & G_{A2}^{\left(C\right)} & = & \left[\begin{array}{llllll} 1 & -1 & 0 & 0 & 1/2 & 1/2 \end{array}\right]\\ L_{A}^{\left(B\right)} & = & \left[\begin{array}{cccccc} 0 & 1 & 0 & \;1/2 & 0 & \;0 \end{array}\;\right] & L_{A}^{\left(C\right)} & = & \left[\begin{array}{llllll} 0 & \;2 & 0 & 0 & 0 & \;0 \end{array}\right] \end{array}$$

which, as with before, do not contain weights for the subject effects. Similarly, the contrast for between-subjects factor B can be derived for both alternative codings as shown in Equation (18).

(18) $$\begin{array}{llllll} G_{B1}^{\left(B\right)} & = & \left[\begin{array}{cccccc} 1 & 1/2 & 1 & \;1/2 & 1/2 & 0 \end{array}\right] & G_{B1}^{\left(C\right)} & = & \left[\begin{array}{llllll} 1 & 0 & \;1 & 0 & 1/2 & \;0 \end{array}\right]\\ G_{B2}^{\left(B\right)} & = & \left[\begin{array}{cccccc} 1 & 1/2 & 0 & \;0 & 0 & 1/2 \end{array}\right] & G_{B2}^{\left(C\right)} & = & \left[\begin{array}{llllll} 1 & 0 & -1 & 0 & 0 & \;1/2 \end{array}\right]\\ L_{B}^{\left(B\right)} & = & \left[\begin{array}{cccccc} 0 & 0 & 1 & 1/2 & 1/2 & -1/2 \end{array}\right] & L_{B}^{\left(C\right)} & = & \left[\begin{array}{llllll} 0 & 0 & \;2 & 0 & 1/2 & -1/2 \end{array}\right] \end{array}$$

which again contain weights for the subject effects and are therefore inappropriate when dealing with software that only implements a single error term.

### 3.3. Contrast Weights for Follow-Up Tests

Another aspect of hypothesis testing in ANOVA models is the use of *post-hoc* contrasts to follow-up omnibus main effects or interaction results. Although often dealt with using *t*-contrasts, most of the discussion in the preceding sections is equivalent for using either *t*- or *F*-contrasts. Indeed, given that *F* = *t*^2^ when rank (**L**) = 1, the discussions in this paper can be taken as equivalent for both the *t* and the *F*. In terms of the actual follow-up tests, the theory remains the same insofar as the *post-hoc* tests of the main effects can be conducted using the same error term as the omnibus test. For interactions, more care must be taken as the error term for the follow-up tests will not necessarily be the same as the error term used for the omnibus effect. As an example, consider following-up the A × B interaction from the model in Equation (9). Using the approach of simple main effects, we may wish to examine the effect of the within-subject factor A at the first level of the between-subjects factor B. The weights for this would be derived as follows

(19) $$\begin{array}{l} {\text{G}}_{A1B1}^{\left(A\right)}=\left[\begin{array}{ccccccccccccc} 1 & 1 & 0 & 1 & 0 & 1 & 0 & 0 & 0 & 1/2 & 1/2 & 0 & 0 \end{array}\right]\\ {\text{G}}_{A2B1}^{\left(A\right)}=\left[\begin{array}{ccccccccccccc} 1 & 0 & 1 & 1 & 0 & 0 & 1 & 0 & 0 & 1/2 & 1/2 & 0 & 0 \end{array}\right]\\ {\text{L}}_{A1-A2\left(B1\right)}^{\left(A\right)}=\left[\begin{array}{ccccccccccccc} 0 & 1 & -1 & 0 & 0 & 1 & -1 & 0 & 0 & 0 & 0 & 0 & 0 \end{array}\right] \end{array}$$

which contains no weights for the subject effects and so can be tested with the overall error term of the model. Alternatively, if we wanted to examine the effect of the between-subjects factor B at the first level of the within-subject factor A, the weights would be

(20) $$\begin{array}{l} {\text{G}}_{A1B1}^{\left(A\right)}=\left[\begin{array}{ccccccccccccc} 1 & 1 & 0 & 1 & 0 & 1 & 0 & 0 & 0 & 1/2 & 1/2 & 0 & 0 \end{array}\right]\\ {\text{G}}_{A1B2}^{\left(A\right)}=\left[\begin{array}{ccccccccccccc} 1 & 1 & 0 & 0 & 1 & 0 & 0 & 1 & 0 & 0 & 0 & 1/2 & 1/2 \end{array}\right]\\ {\text{L}}_{B1-B2\left(A1\right)}^{\left(A\right)}=\left[\begin{array}{ccccccccccccc} 0 & 0 & 0 & 1 & -1 & 1 & 0 & -1 & 0 & 1/2 & 1/2 & -1/2 & -1/2 \end{array}\right] \end{array}$$

which does contains non-zero values for the subject effects. This is perhaps not surprising given that this simple main effect is a between-subject comparison, constrained to only use the estimates from the first level of factor A. Nevertheless, it demonstrates that the error term for the omnibus test may not always be appropriate for testing the simple effects. If one did wish to test this effect, another between-subjects model would need to be specified containing only the data from the first level of the within-subject factor, adding further complication to the approach necessitated by the implementation of the GLM in common neuroimaging packages.

### 3.4. Section Summary

Although contrast weights are a familiar concept for hypothesis testing in the GLM, the inclusion of the random subject effects can make their derivation more difficult depending on the design matrix coding options available. A general approach has been given whereby weights can always be reliably derived using the rows of the design matrix. In addition, this section has shown how contrast weights can provide a complimentary perspective on the issue of suitable error terms. In particular, weights that are derived correctly but contain non-zero values for the subject effects are not suitable for testing with the overall error-term of the model. This provides a useful rule-of-thumb for neuroimaging researchers, particularly when it comes to follow-up tests of interactions, where extra care must be taken given that a suitable error term is not necessarily the same as the error term used for the omnibus effect.

## 4. Building Repeated Measures Models in Neuroimaging Software

Now that the core theoretical concepts of repeated measurements models have been described, we turn to the practical aspect of specifying partitioned-error ANOVA models in neuroimaging software. Based on the discussions in the preceding sections, four generic steps for correctly specifying these models are:

1. Calculate the EMS for the complete model. The number of error terms corresponds to the number of separate models that need to be estimated.
2. For each model, identify which within-subject factors are *not* tested under that error term. Those factors that are missing must be averaged over.
3. Use contrasts at the 1st-level to average-over the various factors identified above.
4. Specify the 2nd-level models using the 1st-level contrasts created in the previous step and then derive the contrast weights using the design matrices.

These steps can be used with any software implementing the mass-univariate GLM approach to modeling group-level neuroimaging datasets. To make these steps clear, an example will now be provided of specifying a 3-way mixed-measures ANOVA using the Flexible Factorial module in SPM12.

### 4.1. Example Data Set

The example data set comes from a previously reported fMRI study by Trotter et al. (2016) investigating the role of serotonin in discriminatory and affective touch perception. Subjects were scanned whilst experiencing gentle stroking of either the arm or the fingers using brushes of different textures. Sixteen of the subjects were administered a tryptophan-depleting amino acid drink prior to the scan, with the remaining 14 subjects receiving a balanced (control) amino acid drink. The design was therefore a 2 × 3 × 2 factorial design with a within-subject factor of *Location* (arm/fingers), a within-subject factor of *Texture* (soft/medium/coarse) and a between-subjects factor of *Drink* (balanced/tryptophan-depleting). It is worth noting that Trotter et al. (2016) conducted the analysis appropriately using the sandwich estimator (SwE) toolbox (Guillaume et al., 2014), however for the purpose of the current paper we shall explore how the data could have been modeled using SPM12 instead.

### 4.2. Example Analysis

The model we wish to fit is given in Equation (6) and is repeated below

$$\begin{array}{ll} y_{ijkl}=\mu & +\alpha_{i}+\beta_{j}+\gamma_{k}+{\left(\alpha \beta \right)}_{ij}+{\left(\alpha \gamma \right)}_{ik}+{\left(\beta \gamma \right)}_{jk}\\ +{\left(\alpha \beta \gamma \right)}_{ijk}+S_{l\left(k\right)}+{\left(S\alpha \right)}_{il\left(k\right)}+{\left(S\beta \right)}_{jl\left(k\right)}+\epsilon_{ijkl} \end{array}$$

In the context of the example dataset, α_*i*_ is the *i*th level of *Location* (*i* = 1, 2), β_*j*_ is the *j*th level of *Texture* (*j* = 1, 2, 3) and γ_*k*_ is the *k*th level of *Drink* (*k* = 1, 2). The indices for the subjects run from *l* = 1, …, *n*_*k*_ where *n*_1_ = 14 and *n*_2_ = 16. As discussed earlier, the *Subject* effects are nested within *Drink* and are considered random-effects.

#### 4.2.1. Step 1: Calculate the EMS and Identify the Number of Models

The EMS for this design have already been derived using Table 5 and are listed in Equation (7). Although the method of Kutner et al. (2004), used to calculate these expressions, is designed for balanced models, it can be applied to unbalanced designs for the purpose of deriving the necessary error terms for each *F*-ratio. The final ANOVA table for this dataset is given in Table 7. The degrees of freedom can be calculated using the rules given in Appendix D.2 of Kutner et al. (2004) and provide one of several ways of checking that each model has been specified correctly in the analysis software. It is also worth mentioning that the EMS can be calculated automatically using the algorithms available in software such as the R package EMSaov (Choe et al., 2017).

**Table 7:** ANOVA table for the example 3-way mixed-measures model.

**Effect**	**Df**
Drink	1
Error: Subject(Drink)	28
Location	1
Location × Drink	1
Error: Subject(Drink) × Location	28
Texture	2
Texture × Drink	2
Error: Subject(Drink) × Texture	56
Texture × Location	2
Texture × Location × Drink	2
Error: Subject(Drink) × Location × Texture	56

#### 4.2.2. Step 2: Identify the Factors to be Averaged-Over

Based on the tests given in Table 7, we can see that there are 4 models needed. The model with the *Subject(Drink)* × *Location* × *Texture* error term uses the original dataset. The model with the *Subject(Drink)* × *Texture* error term requires a data set with the *Location* factor averaged over. Similarly, the model with the *Subject(Drink)* × *Location* error term requires a data set with the *Texture* factor averaged over. Finally, the dataset with only the *Subject(Drink)* error terms requires both the *Location* and *Texture* factors averaged-over for each subject.

To understand why averaging over different effects produces the correct error term, consider the model where we wish to enforce *Subject(Drink)* × *Texture* as the error term. After averaging over the *Location* factor, we can specify the following model

(21) $$\begin{array}{l} y_{.jkl}=\mu +\beta_{j}+\gamma_{k}+{\left(\beta \gamma \right)}_{jk}+S_{l\left(k\right)}+\epsilon_{.jkl} \end{array}$$

Clearly we cannot include any of the terms containing α_*i*_ from the full model, but notice that we cannot include (*Sβ*)_*jl*(*k*)_ either. This is because this term would now be *perfectly collinear* with the errors. Because of this, we know that the error term *is* (*Sβ*)_*jl*(*k*)_. This connects directly with the EMS from Equation (7) where the averaged model has effectively removed the σ^2^ term from EMS_*B*_ and EMS_*BC*_, leaving only $\sigma_{s\beta }^{2}$ as the overall error. Thus the *F*-ratios from the averaged model allow for the effective isolation of the effects of interest.

#### 4.2.3. Step 3: Create the 1st-Level Contrasts for Each of the 2nd-Level Models

The basic 1st-level models for this dataset contain boxcar regressors for each of the *Location* × *Texture* cells of the design. To create the raw data for the *Subject(Drink)* model a single contrast per-subject was specified to average across all the cells. For the *Subject(Drink)* × *Location* model 2 contrasts per-subject were specified, one for each level of *Location* averaged over the levels of *Texture*. For the *Subject(Drink)* × *Location* model 3 contrasts per-subject were specified, one for each level of *Texture* averaged over the level of *Location*. Finally, for the *Subject(Drink)* × *Location* × *Texture* model 6 contrasts per-subject were specified, one for each of the *Texture* × *Location* cells. Of note is the fact that all the condition effects in these contrasts were specified as subtractions from preceding rest periods. This was done to make all images taken to the group-level readily interpretable. This is an important point when implementing repeated measures models with neuroimaging data, given that generally the images taken to the group-level represent within-subject averages, rather than contrasts *per-se*.

#### 4.2.4. Step 4: Specify the Models and Derive the Contrast Weights From the Design Matrices

**Figure 1:**
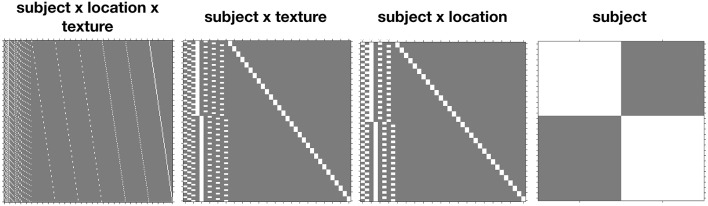
Comparison of the design matrices produced by SPM12 for the different error terms.

Example design matrices for the different models, as specified in SPM12 using the Flexible Factorial module^3^, are given in Figure 1. Once the models have been specified, contrasts for the ANOVA effects can be derived from the design matrix by averaging over the rows after the removal of the subject blocks and reduction to unique-row form^4^. As an example, deriving the weights for testing the *Location* × *Drink* effect from the *Subject(Drink)* × *Location* error model can be achieved in MATLAB using


  
  load('SPM.mat');
  
  % Get design matrix, remove subjects and
    reduce to unique rows
  X = SPM.xX.X;
  X = X(:,1:8);
  X = unique(X, 'rows', 'stable');
  
  % Get weights for the cell means
  A1B1 = mean(X(X(:,1) == 1 & X(:,3) == 1,:));
  A2B1 = mean(X(X(:,2) == 1 & X(:,3) == 1,:));
  A1B2 = mean(X(X(:,1) == 1 & X(:,4) == 1,:));
  A2B2 = mean(X(X(:,2) == 1 & X(:,4) == 1,:));
  
  % Create interaction weights
  L = (A1B1 - A2B1) - (A1B2 - A2B2);
 


where columns 1 and 2 code the levels of *Location* and columns 3 and 4 code the levels of *Drink*.

#### 4.2.5. Results

**Figure 2:**
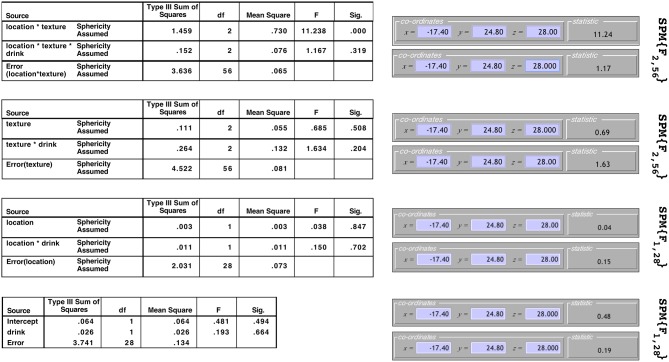
Comparison of the results produced by SPM and SPSS 23 for data from a single voxel. Equivalence of the *F*-statistics and the degrees of freedom confirms that the correct error terms have been selected and that correct Type III weights have been derived.

The results from this model for a single voxel are shown in both SPM and SPSS version 23 (http://www.ibm.com) in Figure 2. Note that to make this comparison valid, all non-sphericity corrections were switched off in SPM (see section 6 for more on this). On the left are the tables from SPSS under the assumption of sphericity and on the right are the test statistic values reported by SPM. The equivalency of the *F*-ratios and degrees of freedom demonstrate how the correct error terms have been selected in SPM and that the contrast weights derived from the design matrix have resulted in the calculation of the correct Type III test statistics.

It is worth noting that although the *F*-ratios are equivalent in Figure 2, the sums-of-squares derived from the models containing averaged datasets will not be the same as those calculated by other statistical software. For instance, consider the *Texture* × *Drink* effect from the *Subject(Drink)* × *Texture* model. In SPSS the sums-of-squares are given as 0.264 for the interaction effect and 4.522 for the error. In SPM these same sums-of-squares are given as 0.132 for the interaction effect and 2.261 for the error. Notice how these both differ by a factor of 2 due to averaging over the two levels of *Location* before fitting the model. Similarly, all the sums-of-squares for the *Subject(Drink)* × *Location* model will be out by a factor of 3, and for the *Subject(Drink)* model will be out by a factor of 2 × 3. Because these differences only amount to a constant in both the numerator and denominator, the *F*-ratios remain the same. As such, this discrepancy is of little concern.

## 5. Use of Continuous Covariates in Repeated Measurement Models

In the previous sections we have seen how complex mixed-measures models can be specified using the GLM framework, as implemented in standard neuroimaging software packages. However, the discussion has so far neglected the formation of ANCOVA models by the inclusion of continuous covariates. Putting aside issues of whether it is meaningful to use certain covariates to “control” for concomitant factors in quasi-experimental situations (see Miller and Chapman, 2001, for discussion), the use of covariates to reduce error variance is an attractive and useful means of increasing the sensitivity of an analysis. This is particularly pertinent for neuroimaging given the general noisiness of the data. Despite this, the use of continuous covariates in classical mixed-measures designs had received limited attention in both the literature and statistical textbooks. Notable exceptions include Federer (1955), Winer (1962), Federer and Meredith (1992), and Federer and King (2007). In particular, the text by Federer and King (2007) contains extensive coverage of this issue and will be used as the basis for the discussion in this section.

### 5.1. The Mixed-Measures ANCOVA Model

The extension of the basic mixed-measures ANOVA model from Equation (3) to a mixed-measures ANCOVA model is given by Federer and King (2007) as

(22) $$\begin{array}{l} y_{ijk}=\mu +\alpha_{i}+\beta_{j}+{\left(\alpha \beta \right)}_{ij}+\beta_{1}{\bar{x}}_{.k\left(j\right)}+\beta_{2}x_{ik\left(j\right)}+S_{k\left(j\right)}+\epsilon_{ijk} \end{array}$$

where *x*_*ik*(*j*)_ is the raw covariate value for repeated measurement *i* from subject *k* in group *j* and ${\bar{x}}_{.k\left(j\right)}$ is the average covariate value for subject *k* in group *j*. In this parameterization, β_1_ gives the between-subject regression slope and β_2_ gives the within-subject regression slope. The inclusion of both regression coefficients is in-line with the recommendations of Federer and King (2007) who state that “in an analysis of covariance…there are as many…regression coefficients as there are error terms in an analysis of variance…” (p. 240). Using this approach, the between-subjects effects are adjusted for β_1_ and the within-subject effects are adjusted for β_2_. The model in Equation (22) is therefore the basis for any mixed-measures model that contains a continuous covariate. Implementation will largely depend on whether the covariate in question is measured between-subjects or within-subject, as will be discussed below.

#### 5.1.1. Between-Subjects Covariates

A between-subjects covariate (also known as *constant* or *time-invariant*) is defined based on having a single value per-subject that does not depend on the within-subject manipulation. The inclusion of a between-subjects covariate in Equation (22) renders the *x*_*ik*(*j*)_ term redundant and the model can be simplified to only contain the *x*_.*k*(*j*)_ term. Note that when using the multiple-model approach advocated in the previous section, the covariate must be tested within the same model as the other between-subject effects and interactions (i.e., the *Subject(Drink)* model from the previous example). All other models should contain the covariate (by replicating the per-subject values), but should not be used for testing the effect. The only exception is when an interaction between the covariate and a within-subject factor is included. In this instance, the interaction effect would be tested within the same model as the test of the interacting within-subject factor. Inclusion of these interactions is a modeling choice that provides equivalence with the repeated measurement models generated by software such as SPSS, as well as providing equivalence with the multivariate approach to repeated measures (see McFarquhar et al., 2016, for details).

#### 5.1.2. Within-Subject Covariates

A within-subject covariate (also known as *time-varying* or *time-dependent*) is defined based on having multiple values per-subject that depend on the within-subject manipulation. Unlike a between-subject covariate, there are no redundancies in Equation (22) and both terms are therefore necessary. Furthermore, when implementing the multiple-model approach from the previous section, it is important to include as many of the covariates as possible in each model. For some of the models, averaging over certain factors will create redundancies across the covariates that can be removed. For instance, the model used to test the between-subject effects will only contain ${\bar{x}}_{.k\left(j\right)}$, whereas the model used to test the within-subject main effect and interaction can contain both ${\bar{x}}_{.k\left(j\right)}$ and *x*_*ik*(*j*)_. In a similar vein to testing the traditional ANOVA effects, the parameter associated with ${\bar{x}}_{.k\left(j\right)}$ should be tested using the between-subject error term and the parameter associated with *x*_*ik*(*j*)_ should be tested using the within-subject error term.

As a more involved example, consider an ANCOVA for the complete 2 × 3 × 2 mixed-measures design from section 4. Assuming there is a covariate value per-cell of the design, extension of the *split block* ANCOVA model presented in Federer and King (2007) provides the model form

(23) $$\begin{array}{ll} y_{ijkl}=\mu & +\alpha_{i}+\beta_{j}+\gamma_{k}+{\left(\alpha \beta \right)}_{ij}+{\left(\alpha \gamma \right)}_{ik}+{\left(\beta \gamma \right)}_{jk}+{\left(\alpha \beta \gamma \right)}_{ijk}\\ +\beta_{1}{\bar{x}}_{..l\left(k\right)}+\beta_{2}{\bar{x}}_{i.l\left(k\right)}+\beta_{3}{\bar{x}}_{.jl\left(k\right)}+\beta_{4}x_{ijl\left(k\right)}\\ +S_{l\left(k\right)}+{\left(S\alpha \right)}_{il\left(k\right)}+{\left(S\beta \right)}_{jl\left(k\right)}+\epsilon_{ijkl} \end{array}$$

where *x*_*ijl*(*k*)_ is the raw covariate value, ${\bar{x}}_{..l\left(k\right)}$ is the average covariate value for subject *l* from group *k*, ${\bar{x}}_{i.l\left(k\right)}$ is the average covariate value for subject *l* from group *k* from level *i* of the first within-subject factor and ${\bar{x}}_{.jl\left(k\right)}$ is the average covariate value for subject *l* from group *k* from level *j* of the second within-subject factor. The ANOVA table for this model, indicating the most suitable error terms for testing the covariates, is given in Table 8.

**Table 8:** ANOVA table for the example 3-way mixed-measures model including a within-subject covariate.

**Effect**	**Df**
Covariate (${\bar{x}}_{..l\left(k\right)}$)	1
Drink	1
Error: Subject(Drink)	27
Covariate (${\bar{x}}_{i.l\left(k\right)}$)	1
Location	1
Location × Drink	1
Error: Subject(Drink) × Location	27
Covariate (${\bar{x}}_{.jl\left(k\right)}$)	1
Texture	2
Texture × Drink	2
Error: Subject(Drink) × Texture	54
Covariate (*x*_*ijl*(*k*)_)	1
Texture × Location	2
Texture × Location × Drink	2
Error: Subject(Drink) × Location × Texture	54

An additional complication arises when one of the covariates in Equation (24) is associated with one within-subject factor, but is constant over the other. In this situation there will be redundancies in the definitions of the four covariates. For instance, if one of the within-subject factors was time and a value was measured only once per-visit, the value would be constant across any other within-subject variables. Implementation of this design would then be similar in spirit to the use of between-subject covariates in Equation (22), insofar as all covariates terms would attempt to enter each model, but would then be dropped wherever redundancies are found.

## 6. The Assumption of Sphericity

One final important issue to discuss is the much-maligned sphericity assumption of the traditional repeated-measures ANOVA. In brief, the validity of the *F*-ratios, in terms of following an exact *F*-distribution under the null, is predicted on assuming a spherical structure to the variance-covariance matrix. This can be expressed as

(24) $$\begin{array}{l} \text{Var}\left(y_{i}-y_{j}\right)=\sigma_{i}^{2}+\sigma_{j}^{2}-2\sigma_{ij}=\lambda \;\forall i\neq j \end{array}$$

which indicates that for all pairs of measurements the variance of their differences are identical. This is therefore a similar (but less restrictive) case of compound symmetry (Davis, 2002).

Traditionally, departures from sphericity are assessed using hypothesis tests, such as described by Mauchly (1940). If significant departures from sphericity are found then corrections to the degrees of freedom, such as those after Greenhouse and Geisser (1959) or Huynh and Feldt (1976), can be applied. Generally speaking, neuroimaging software does not implement such corrections. For instance, use of the OLS algorithm in FSL means assuming sphericity for the validity of the *F*-tests at every voxel. Use of permutation tests via Randomize (Winkler et al., 2014) do not have such restrictive assumptions, although the exchangeable structure of the data is more complex and must be accommodated for accurate derivation of the null distribution (see Winkler et al., 2015, for details). SPM, on the other-hand, implements a correction for departures from sphericity, but there are some caveats. Firstly, the covariance matrix used to derive the correction comes from a subset of pooled voxels, rather than being applied at each voxel individually. As discussed by McFarquhar et al. (2016), the method used to select voxels to enter this pool can have a dramatic effect on the number of voxels that subsequently survive correction. Secondly, the impact of this correction on the specification of repeated measurement models remains unclear. According to Glaser and Friston (2007), the non-sphericity correction employed by SPM is essentially a whitening procedure that renders the covariance structure a scalar multiple of an identity matrix. This is therefore equivalent to the method of generalized least-squares (e.g., Faraway, 2016), which can be used to specify a marginal model that accommodates a variety of covariance structures without the need for random effects (see Guillaume et al., 2014). The implication here would seem to be that when the non-sphericity procedure is employed the inclusion of the random subject blocks is unnecessary. However, the current default SPM implementations of both the paired *t*-test and one-way within-subject ANOVA make use of the non-sphericity correction with random subject blocks. At present it is unclear why this is the case and requires further clarification from the SPM authors.

## 7. Conclusions

This paper has discussed the modeling of group-level repeated measurements in neuroimaging, using the traditional GLM framework. The core statistical concept of the EMS has been discussed and from these discussion a set of steps for implementing these forms of models in software have been given. Additional considerations, such as covariates and the assumption of sphericity, have also been discussed. The main conclusion from this paper is that if one wishes to use traditional neuroimaging analysis tools for this purpose, great care must be taken to correctly derive the tests from the EMS and then to carefully implement the multiple models necessitated by the GLM framework. In doing so, it is important to carefully consider the contrasts used and the error-terms employed, especially for follow-up tests from interaction effects. To that end, the oft-quoted advice given by Gläscher and Gitelman (2008) should no longer be relied-upon for deriving contrast weights as it may lead to inappropriate tests of the model effects. Furthermore, the example given in this paper has highlighted how tedious and complicated the implementation of these models can be in standard software. Ultimately, there are much better alternative tools available for this purpose. Examples include the author's own multivariate and repeated measures (MRM) toolbox (McFarquhar et al., 2016), the previously mentioned SwE toolbox (Guillaume et al., 2014), the AFNI tool 3dMVM (Chen et al., 2014), and the mixed-effects approaches discussed by Chen et al. (2013). There are also tools available which seek to simplify the specification of the traditional partitioned-error ANOVA, such as GLMFlex (McLaren et al., 2011), although the limitations of the traditional ANOVA framework should be enough for researchers to consider the more modern alternatives mentioned above. McFarquhar et al. (2016) provides comparison between several of these tools (including MRM, SwE, and GLMFlex) noting that largely their differences come down to assumptions about the covariance structure of the data, available methods for multiple-comparison correction, the ability to accommodate certain features of the data (e.g., missing data, within-subject covariates) and the user-friendliness of the implementation.

In terms of a more general conclusion from this paper, it is important for developers of neuroimaging analysis packages to recognize that the onus of correctly specifying these models should not be placed on the users. Indeed, considering the methods outlined in this paper it would seem wholly unfair to expect that users would know to perform the given steps without any clear guidance. Instead, software developers should strive for improved usability and clarity in their implemented methods. Although usability is not always considered as carefully compared with commercial software, it is hopefully clear that only by providing user-friendly and well-documented software can the neuroimaging community be confident in the accuracy of their methods. This is particularly true given recent concerns about the accuracy of common neuroimaging analysis approaches (Eklund et al., 2016) as well as more general concerns about the replicability of behavioral research (Open Science Collaboration, 2015). At present it is unclear how many published neuroimaging analyses were conducted using inappropriate methods of analysing repeated measurements. What is clear is that neuroimaging needs a renewed focus on the methods and software that are readily employed to analyse brain imaging data. This is the only way for the neuroimaging community to have faith that published analyses have been implemented correctly and provide results that can be interpreted accurately.

## Author Contributions

The author confirms being the sole contributor of this work and has approved it for publication.

### Conflict of Interest Statement

The author declares that the research was conducted in the absence of any commercial or financial relationships that could be construed as a potential conflict of interest.

## Footnotes

^1^It is notable that AFNI (https://afni.nimh.nih.gov) has facilities included to accommodate random effects in the group-level ANOVA programs 3dANOVA2 and 3dANOVA3, as well as facilities for multivariate approaches to repeated-measurements in 3dMVM. As such, the discussions in the paper are less relevant for AFNI users.

^2^Be aware that this approach will not work with unbalanced design matrices. See section 4.2.4 for how this procedure can be adjusted for those cases.

^3^For the current release of SPM12, it is necessary to alter the spm_cfg_factorial_design.m file (inside /spm12/config) and change the line fnums.num = [2 1] to fnums.num = [Inf 1] to allow for interactions higher than a two-way.

^4^Removal of the subject blocks allow for derivation of Type III sums-of-squares in unbalanced designs by reducing the design matrix to a balanced unique-row form. This is based on assuming that the effect in question is being testing within an appropriate model and thus the weights on the subject effects will be zero. If this is not the case, then a non-estimable contrast will be returned.
